# Role of FK506-sensitive signals in asthmatic lung inflammation

**DOI:** 10.3389/fimmu.2022.1014462

**Published:** 2022-11-09

**Authors:** Chihiro Tomiaki, Kosuke Miyauchi, Sewon Ki, Yoshie Suzuki, Narumi Suzuki, Hiroshi Morimoto, Yohei Mukoyama, Masato Kubo

**Affiliations:** ^1^ Laboratory for Cytokine Regulation, Research Center for Integrative Medical Sciences (IMS), RIKEN Yokohama Institute, Yokohama, Kanagawa, Japan; ^2^ Medical Affairs Department, Maruho Co., Ltd., Osaka, Japan; ^3^ Global Business Development Department, Maruho Co., Ltd., Kyoto, Japan; ^4^ Division of Molecular Pathology, Research Institute for Biomedical Science, Tokyo University of Science, Chiba, Japan

**Keywords:** asthma, innate lymphocyte cells (ILCs), Th2 airway inflammation, cytokines, IL-13

## Abstract

Asthma is airway inflammatory diseases caused by the activation of group 2 innate lymphoid cells (ILC2s) and type 2 helper T (T_H_2) cells. Cysteine proteases allergen cause tissue damage to airway epithelial cells and activate ILC2-mediated type 2 airway inflammation. FK506 is an immunosuppressive agent against calcium-dependent NFAT activation that is also effective against asthmatic inflammation. However, the effects of FK506 on cysteine protease allergen-mediated airway inflammation remain unclear. In this study, we investigated the suppressive effects of FK506 on airway inflammation. FK506 had a partial inhibitory effect on ILC2-dependent eosinophil inflammation and a robust inhibitory effect on T cell-dependent eosinophil inflammation in a cysteine protease-induced mouse asthma model. The infiltration of T1/ST2^+^ CD4 T cells in the lungs contributed to the persistence of eosinophil infiltration in the airway; FK506 completely inhibited the infiltration of T1/ST2^+^ CD4 T cells. In the initial phase, FK506 treatment targeted lung ILC2 activation induced by leukotriene B_4_ (LTB_4_)-mediated calcium signaling, but not IL-33 signaling. FK506 also inhibited the IL-13-dependent accumulation of T1/ST2^+^ CD4 T cells in the lungs of the later responses. These results indicated that FK506 potently suppressed airway inflammation by targeting ILC2 activation and T1/ST2^+^ CD4 T cell accumulation.

## Introduction

Asthma is a lifelong disorder usually driven by type 2 immune-inflammatory pathogenic mechanisms. The inflammatory responses observed in asthma are complex, and airway epithelial cells (AECs) are critical for the initial development of local inflammation (1). Several allergens, including house dust mites, food, and fungi, possess group 1 cysteine protease activity, which increases the permeability of local epithelial cells. Tissue damage caused by cysteine proteases allergen allows the release of epithelial-derived cytokines, interleukin (IL)-33, IL-25, and thymic stromal lymphopoietin (TSLP), which activate group 2 innate lymphocytes (2). Damaged AECs release IL-33 and TSLP, basophils produce IL-4, and T_H_2 cells produce type 2 cytokines, including IL-4, IL-5, and IL-13, which mainly contribute to allergic airway inflammation, subsequently leading to allergen-specific IgE-mediated mast cell degranulation and eosinophil recruitment (3–5). ILC2s secrete relatively high amounts of IL-5 and IL-13 (6–8). These ILC2-derived type 2 cytokines also play critical roles in hyperplasia, mucin formation in bronchial epithelial cells, and eosinophil accumulation (9, 10). ILC2-derived type 2 cytokines could also contribute to the accumulation of T_H_2 cells in the local inflammation site (10–12). Recently, inflammatory mediators synthesized from arachnoid acids, such as prostaglandin D_2_ (PGD_2_) and leukotriene D_4_ (LTD_4_), have been reported to activate ILC2s (13, 14). Therefore, cysteine proteases allergen promote several aspects of asthmatic airway inflammation by activating innate immune responses.

Several treatments are generally used to control and prevent asthma attacks. Systemic treatment with steroids, such as corticosteroids, is ineffective in some patients with severe asthma (15). Moreover, the long-term use of steroids induces side effects (16). Leukotriene modifiers are a promising treatment to control asthma since they block the actions of leukotrienes, which tighten the airway muscles (17). An immunosuppressive agent, FK506, blocks calcium-dependent nuclear factor of activated T cells (NFAT) activation, and T-cell activation (18–20) is another promising treatment for asthma by targeting type 2 cytokine release from T_H_2 cells (21, 22). In a mouse model of *Aspergillus*-induced asthma, FK506 targets chronic asthmatic inflammation, improving eosinophil infiltration (23). However, the effects of FK506 on ILC2 activation remain controversial. IL-33-dependent ILC2 activation is expected to be resistant to FK506, whereas lipid mediator-mediated activation *via* LTB_4_, LTD_4_, and LTE_4_ is expected to be susceptible (24). Therefore, the precise effect of FK506 on the cysteine protease allergen-mediated airway inflammation remains unclear. In particular, it is unclear whether innate or adaptive immune responses are the primary targets of FK506 treatment.

In this study, we investigated the mechanism underlying the inhibitory effect of FK506 on cysteine protease allergen-induced airway inflammation in mice. We found that FK506 effectively inhibited ILC2 activation and ST2^+^ CD4 T cells accumulation in the lung. ILC2s are the initial target of FK506, inhibiting their functions *via* calcium-dependent activation, including LTB_4-_induced activation. These findings indicate that ILC2 and CD4 T cells are potential targets of FK506 and shed light on the mechanisms that inhibit airway inflammation induced by protease allergens.

## Results

### FK506 inhibits protease allergen-induced acute phase airway inflammation

**Figure 1:**
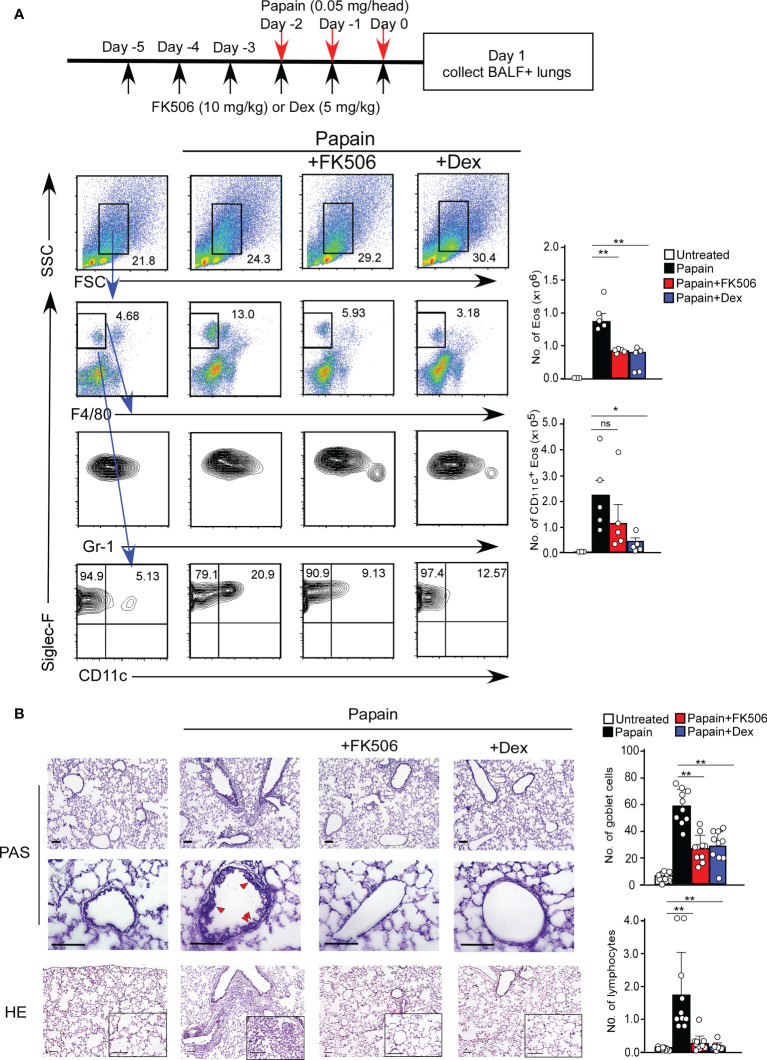
**(A)** The papain and FK506 or Dexamethasone (Dex) treatment protocol is presented by the schematic diagram (top). The mice were administrated with (black bars, n=5) or without (white bars, n=5) papain. Papain-administrated mice were further treated with FK506 (red bars, n=8) or Dex (blue bars, n=5). Lung cells were prepared from the lung homogenate on day one after the final papain injection (see Materials and Methods). Flow cytometry analysis assessed the percentage (left flow profiles) and cell numbers (right graphs) of Siglec-F+ and CD11c+ eosinophils (Eos). **(B)** PAS (top: low magnification; middle: high magnification) and HE (bottom) staining of lung sections from FK506- or Dex-treated mice. Red arrow heads indicate mucus glycans positive goblet cells. Goblet cells and lymphocytes were counted in 200 µm × 200 µm regions in each lung section (bar graphs, n=10). Bars represent the means; not significant (ns), *p–0.05, **p–0.01 using unpaired Mann-Whitney U-tests. All error bars represent SEM. Scale bars, 100 µm. FACS and section dates are representative of three experiments.

We first investigated the effect of FK506 on T cell-independent airway inflammation caused by protease allergens in a papain-induced mouse model. Intranasal administration of papain for three days generated goblet cell hyperplasia and caused the infiltration of activated eosinophils in the lungs 24 h after the third injection. Papain treatment significantly increased eosinophil infiltration compared to PBS-treatment (p<0.01). In contrast, the intraperitoneal administration of FK506 partially reduced the number of infiltrating lung eosinophils (p<0.01) (**Figure 1A**) and significantly inhibited goblet cell generation and lymphocyte accumulation (**Figure 1B**). These inhibitory effects of FK506 were comparable to or slightly lower than those of dexamethasone, a corticosteroid (**Figures 1A, B**). These results indicate that FK506 inhibits protease-induced airway inflammation.

**Figure 2:**
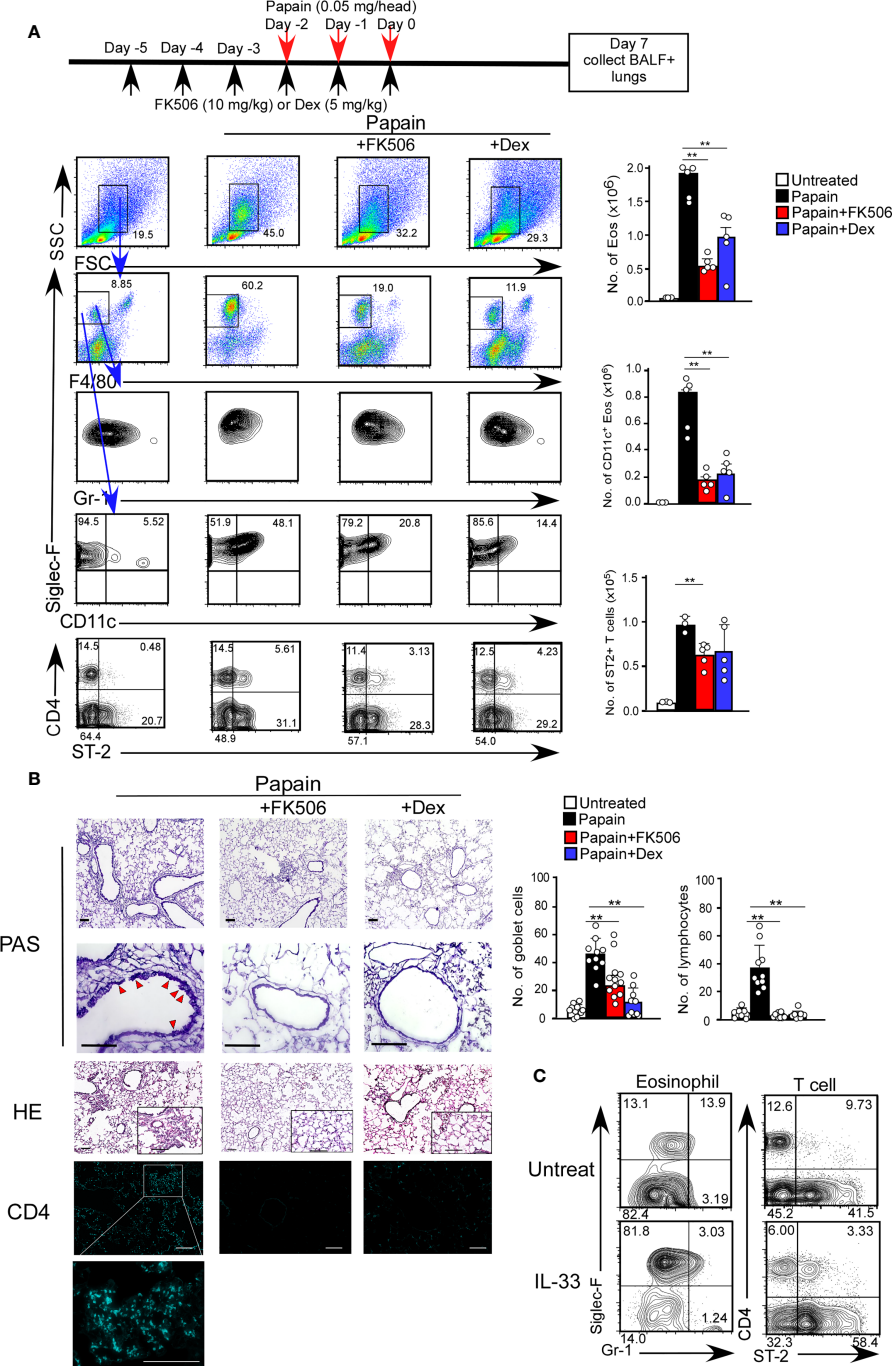
FK506 inhibited protease allergen-induced T cell-mediated airway inflammation. **(A)** The papain and FK506 or Dex treatment protocol is presented by the schematic diagram (top). Lung cells from papain-administrated mice (black bars, n=5) treated with FK506 (red bars, n=5) or Dex (blue bars, n=5) were harvested on day seven. **(B)** PAS^+^ Goblet cells (top: low magnification; second from top: high magnification) and Eos (HE, second from bottom), and CD4^+^ T cells (bottom, right blue) were stained in lung sections at day seven, and were analyzed by fluorescence microscopy (low magnification, x20; high magnification, x100; Scale bars, 100 µm). Goblet cells and lymphocytes were counted in 200 µm × 200 µm regions in each lung section (bar graphs, n=10). Red arrow heads indicate mucus glycans positive goblet cells. **(C)** The mice were administrated with mrIL-33 (1 µg/head/day). Lung cells were harvested on day four, and the proportions (left flow profiles) and cell numbers (right graph) of Siglec-F^+^ and CD11c^+^ Eos and ST-2^+^CD4^+^ T cells were assessed. Bars represent means; **p<0.01 using unpaired Mann-Whitney U-tests. All error bars represent SEM. FACS and section data are representative of three experiments.

The acute airway response is T cell-independent and largely depends on innate immune cells, including basophils and ILC2s (25). Interestingly, the infiltration of CD11c^+^ eosinophils in the lungs was sustained seven days after the initial papain treatment (**Figure 2A**). The persistence of eosinophil migration might influence the migration of CD4 T cells expressing the IL-33 receptor T1/ST2, which was not observed on day one (**Figure 2A**).

To further investigate the effect of FK506 on the day seven response, we analyzed the inhibitory effect of FK506 on the persistent activation of eosinophils on day 7. FK506 treatment attenuated the papain-induced infiltration of activated CD11c^+^ eosinophils and accumulation of T1/ST2^+^ CD4 T cells into the lungs (**Figure 2A**). Interestingly, the effect of dexamethasone on T cell migration was more subtle than that of FK506 (**Figure 2A**). Moreover, FK506 also inhibited papain-induced goblet cell hyperplasia and lymphocyte accumulation (**Figure 2B**). These results suggest that FK506 also inhibits the T cell-mediated persistence of eosinophil attraction. Similar T1/ST2^+^ T cell migration with eosinophil attraction was recapitulated in mice nasally injected with IL-33 alone (**Figure 2C**), indicating that IL-33 receptor-expressing CD4 T cells, contributed to the persistence of eosinophil attraction.

**Figure 3:**
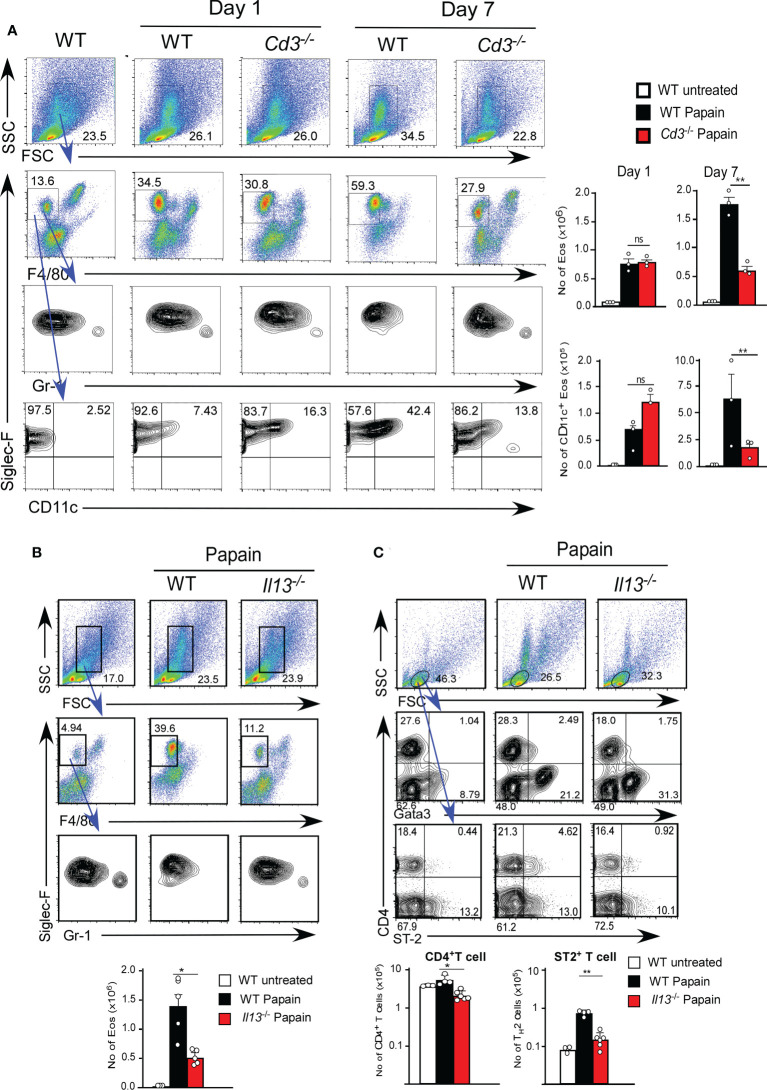
**(A)** Cd3-sufficient (WT, n=3) or -deficient (n=3) mice were administrated with papain. Lung cells were harvested on day one or seven after the final papain injection, and the proportions and cell numbers of Eos and T cells were assessed. **(B, C)** Il13-sufficient (WT, n=5) or -deficient (n=5) mice were administrated with papain. The proportions (left flow profiles) and numbers (right graphs) of Eos **(B)** and ST-2+CD4+ T cells **(C)** were assessed at seven days. Bars represent the means; not significant (ns), *p–0.05, **p–0.01 using unpaired Mann-Whitney U-tests. All error bars represent the SEM. Data are representative of three **(A, B)** or two **(C)** experiments.

Thus, we examined the role of T1/ST2^+^ T cell cells using T cell-deficient mice (*Cd3* ^-/-^ mice). *Cd3* ^-/-^ mice showed papain-induced eosinophil infiltration on day one but did not show persistent infiltration of activated eosinophils on day seven (**Figures 3A**). These results indicate that T cells play a critical role in the continuous activation of eosinophils, supporting our hypothesis that the infiltration of T1/ST2^+^ CD4 T cells contributes to the persistence of papain-induced airway inflammation.

IL-13 secreted by ILC2s is thought to be necessary for the development of T_H_2 cells by modifying DC2 function (6). We next tested whether IL-13 plays a role in T1/ST2^+^ CD4 T cell-dependent eosinophil attraction in IL-13-deficient mice (*Il13^Tomato/Tomato^*). IL-13-deficient mice exhibited a marked reduction in papain-induced T1/ST2^+^ CD4 T cell attraction along with a decrease in the persistent infiltration of eosinophils (**Figure 3B**). T cells that emerged in the lung after papain treatment expressed the transcription factor GATA3, which is predominantly expressed by T_H_2 cells (**Figure 3C**). These results suggested that the T1/ST2^+^ CD4 T cells constitute a subset of T_H_2 cells. Therefore, we speculate that T1/ST2^+^ T_H_2 attraction into the lung controls the persistence of papain-induced airway inflammation and that FK506 inhibits T_H_2 cell attraction by impairing ILC2 activation.

### Inhibitory effect of FK506 on ILC2 activation

**Figure 4:**
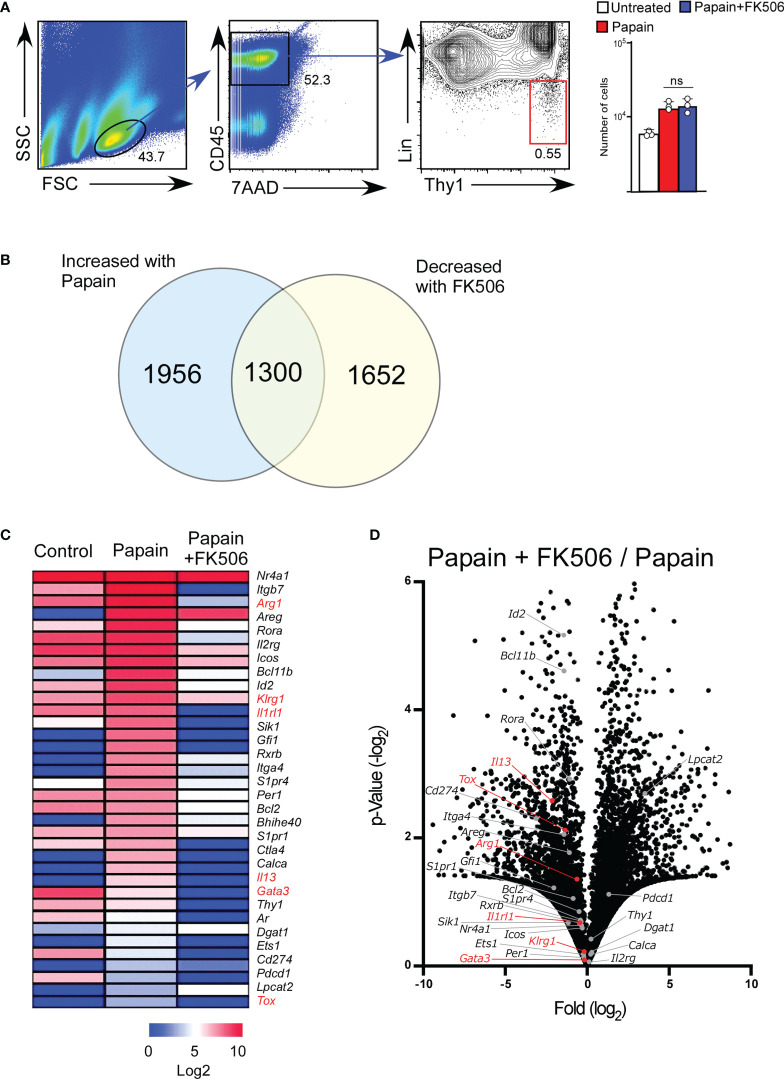
FK506 inhibits the papain-induced activation signal of ILC2 cells. Total RNA was isolated from lung ILC2 cells on day one after papain administration. We compared the results with and without FK506 treatment, as shown in **Figure 1**. RNAseq libraries were sequenced using the HiSeq platform. **(A)** Gating strategy and the number of lung ILC2 cells (untreated, n=3; papain-administered with or without FK506 treatment, n=3 each). CD45^+^ lineage marker (Lin)-Ty1^+^ ILC2 cell populations are marked with a red square. **(B)** Venn diagram of genes whose expressions were increased by papain (>2-fold) and decreased by FK506 (<2-fold). **(C)** Heat maps representing the FPKM values of ILC2 signature genes in lung ILC2 cells. The data indicate untreated (control) and papain-administered mice with (Papain + FK506) or without (Papain) FK506. **(D)** Volcano plots represent the fold-change expression (horizontal axis) and p-values (vertical axis) in the papain versus papain + FK506 comparison. ILC2 signature genes are shown in gray (n=3). ILC2 activation marker genes (Arg1, Gata3, Il13, Tox, Klrg1, and Il1rl1) are highlighted in red. Data are representative of four mice. Bars represent the means; not significant (ns) using unpaired Mann-Whitney U-tests.

**Figure 5:**
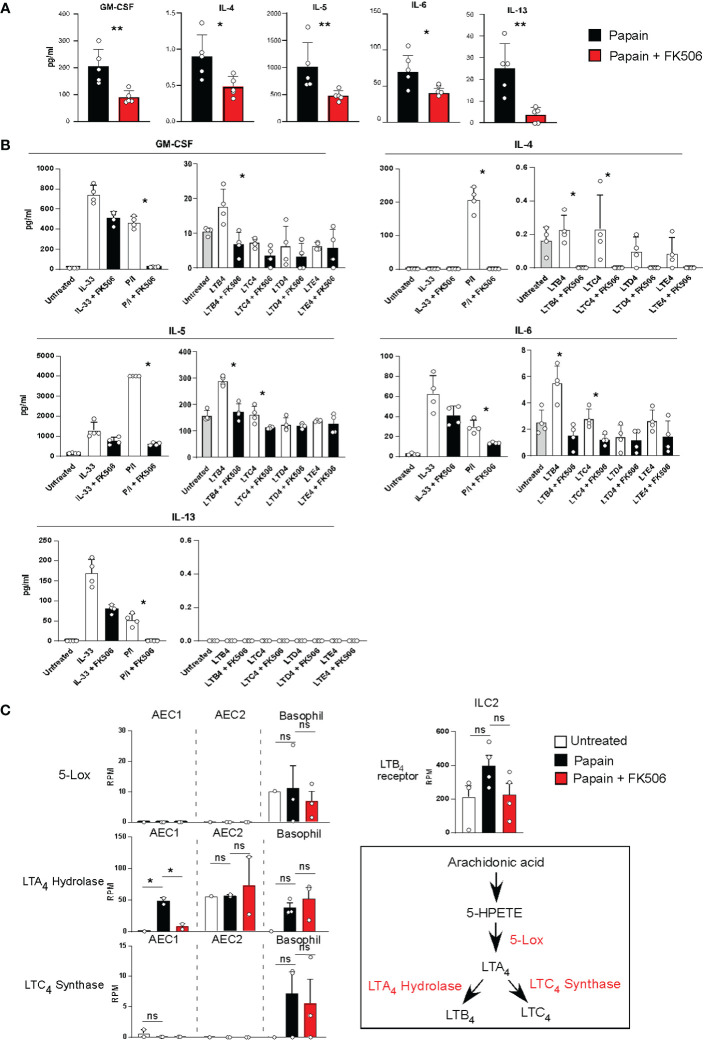
**(A)** Lung ILC2 cells were isolated from papain-administrated mice treated with (n=5) or without (n=5) FK506 and were cultured in the presence of rIL-7 for 12 h. Cytokine and chemokine levels in the cell supernatants were measured using MAGPIX System. **(B)** Pooled ILC2 cells were isolated from the lung of 20 unstimulated mice. Ten thousand cells were cultured on a 96-well plate in the presence of rIL-7 for 40 h. The cells were activated with LTs, IL-33, or PMA + ionomycin treated with (n=5) or without FK506 (n=5). Levels of type 2 cytokines were assessed as described above. **(C)** The left panel indicates the expression of lipid mediator genes in AEC1 cells (n=3), AEC2 cells (n=3), and basophils (n=3). The right-top panel indicates the expression of the LTB4 receptor in lung ILC2 cells as described in Figure 4. The right-bottom panel represents the biosynthetic pathway of LTB4. Bars represent the means; not significant (ns), *p–0.05, **p–0.01 using unpaired Mann-Whitney U-tests. All error bars represent the SEM.

To examine the effect of FK506 on ILC2 activation, we performed RNA-seq analysis of lung ILC2 cells from mice nasally administered papain with or without FK506. Although FK506 treatment had no effect on the total number of ILC2s in the lungs (**Figure 4A**), FK506 treatment inhibited 1,300 genes whose expression increased >2-fold in response to papain. These FK506-sensitive genes included several ILC2 signature genes, such as arginase 1 (*Arg1*), *Gata3*, *Tox*, *Il13*, *Klrg1*, and *Il1r1* (**Figures 4B–D**; **Supplementary Figure 1**). These results suggest that the inhibitory effect of FK506 on T_H_2 cell attraction is due to the inhibition of IL-13 and ILC2 activation in the papain-induced asthmatic responses. To investigate the effect of FK506-mediated suppression on papain-induced ILC2 activation, we measured the protein expression of ILC2-derived cytokines. Papain administration enhanced the production of GM-CSF and several type 2 cytokines (**Figures 5A, B**). Interestingly, papain, but not IL-33, induced detectable levels of IL-4 in activated ILC2 cells (**Figure 5B**). FK506 markedly inhibited the production of GM-CSF, IL-5, and IL-13 in papain-activated ILC2 cells (**Figure 5A**). These results suggested that the FK506-mediated inhibition of T_H_2 infiltration could be due to the suppression of IL-13, and that FK506 possibly inhibited eosinophil activation by suppressing GM-CSF and IL-5 expression.

The next question concerns the role of FK506-sensitive calcium signals in ILC2 activation, which lead to eosinophil accumulation in the lungs. It has been reported that AEC-derived IL-33 activates ILC2s, IL-25, TSLP, basophil-derived IL-4, neuropeptides, and lipid mediators, including leukotrienes (LTs) (24–27), and that LTs provide calcium signals to induce IL-4 production (28). **Figure 5A** indicates that papain treatment triggered detectable levels of IL-4 in ILC2 cells. Therefore, we examined LT synthesis as a stimulus for ILC2 activation in this study. Naïve ILC2 cells were stimulated with cysteinyl leukotriene B_4_ (LTB_4_), C_4_ (LTC_4_), D_4_ (LTD_4_), or E_4_ (LTE_4_) in the presence or absence of FK506. ILC2 cells were also stimulated with IL-33 or PMA + ionomycin as prototypic calcium-independent or -dependent stimuli. LTB_4_ provides an activation signal to induce GM-CSF, IL-5, and IL-13 production in ILC2 cells. FK506 markedly inhibited cytokine production caused by PMA+ionomycin and LTB_4_ (**Figure 5B**), suggesting that FK506-sensitive calcium signals largely contributed to the production of GM-CSF and type 2 cytokines during papain-induced ILC2 activation.

We further examined transcriptomic changes in the LTB_4_ pathway in basophils and type I and II alveolar epithelial cells (AEC1 and 2) (**Supplementary Figure 2**). RNA-seq analysis indicated that 5-LOX was constantly expressed in basophils but not in AECs. Moreover, LTB_4_-synthesizing enzymes and leukotriene A_4_ (LTA_4_) hydrolase were consistently expressed in AEC1, AEC2, and basophils. FK506 inhibited the papain-induced LTA_4_ hydrolase expression in AEC1, whereas their expressions in AEC2 and basophils were resistant to FK506 (**Figure 5C**). Moreover, FK506 seemed to be sensitive in the expression of receptors against LTB_4_ in ILC2 cells, suggesting that LTB_4_ signaling is a target of the FK506-mediated inhibition of ILC2 activation. In addition, we performed IPA pathway analysis of the AEC1, AEC2, and basophil transcriptome data but were unable to uncover other pathways involved in ILC2 activation (**Supplementary Figure 2**). Thus, we conclude that the LTB_4_ pathway might be important for ILC2 activation pathway in papain-induced allergic responses and that it constitutes a putative target of the FK506-mediated inhibition of these responses. However, more studies should be conducted in order to fully validate this hypothesis.

### FK506 did not inhibit IL-33-induced eosinophil attraction

**Figure 6:**
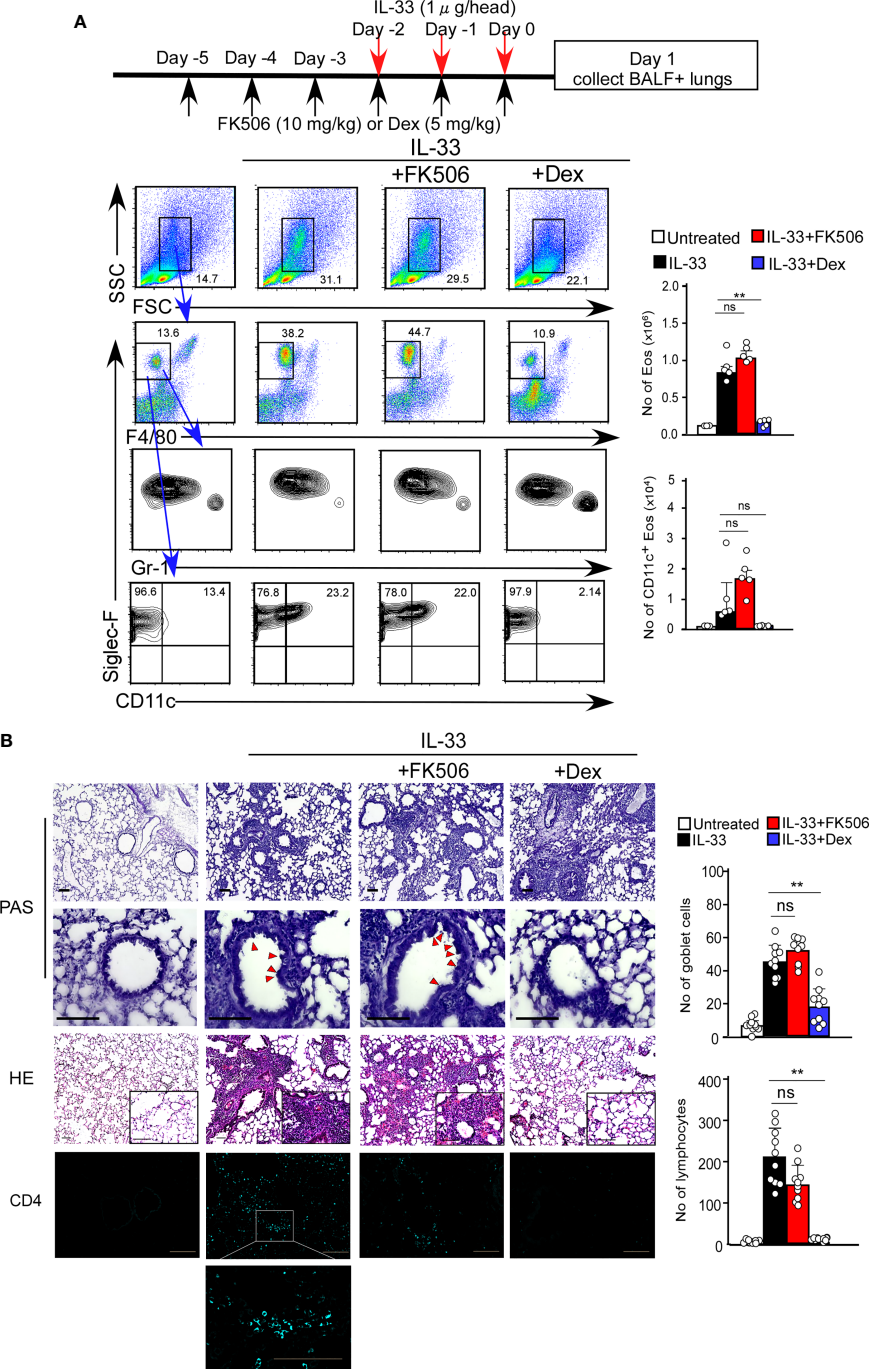
**(A)** The IL-33 and FK506 or Dex treatment protocol is presented by the schematic diagram (top). IL-33-administered mice (black bars, n=5) were treated with FK506 (red bars, n=5) or Dex (blue bars, n=5). Lung cells were harvested on day one after the final papain administration. The data indicated the percentages (left flow data) and the numbers (right graphs) of Eos. **(B)** PAS (top: low magnification; second from top: high magnification), HE (second from bottom) and CD4 (bottom, right blue) staining of the lung sections, and were analyzed by fluorescence microscopy (low magnification, x20; high magnification, x100; Scale bars, 100 µm). Goblet cells and lymphocytes were counted in 200 µm × 200 µm regions in each lung section (bar graphs, n=10). Red arrow heads indicate mucus glycans positive goblet cells. Bars represent the means; not significant (ns), **p–0.01 using unpaired Mann-Whitney U-tests. All error bars represent the SEM. Data are representative of three experiments.

Tissue damage to the airway epithelium caused by papain-mediated protease activity allows the release of IL-33, which promotes ILC2 activation. Interestingly, epithelial-derived IL-33, which is expressed explicitly in AEC2 cells, was constantly expressed at high levels irrespective of FK506 treatment (**Supplementary Figure 1**). However, the levels of the IL-33 receptor (*Il1rl1*) in ILC2 cells were reduced by FK506 (**Figure 4C**). Therefore, we assumed that the pathways downstream of IL-33 signaling are possible targets of FK506. To evaluate this, we investigated the effects of FK506 on IL-33-induced airway inflammation. The intranasal administration of IL-33 resulted in the marked accumulation of activated eosinophils, even in the absence of papain (**Figure 6A**). In addition, IL-33 promoted the hyperplasia of goblet cells and the attraction of T1/ST2^+^ T_H_2 cells (**Figure 6B**). Dexamethasone attenuated IL-33-induced airway inflammation; however, FK506 failed to inhibit IL-33 function (**Figures 6A, B**). These results suggest that FK506 mainly targets type 2 inflammation, which is controlled by the IL-33-independent activation of ILC2 cells.

## Discussion

FK506, which targets calcium signaling, is a promising therapeutic agent for airway inflammatory diseases (21, 29, 30). Here, we demonstrated that FK506 dampened the induction of an asthmatic inflammatory response by inhibiting two pathways: the activation of lung ILC2 cells and the accumulation of T1/ST2^+^ T_H_2 cells. In the T cell-independent induction phase, the calcium signal of ILC2 cells was a primary target of FK506. In contrast, the induction of T_H_2 cells was the second target at later responses, which migrate into the lung and persist during airway inflammation. Our analyses of a cysteine protease allergen-induced mouse model of airway inflammation demonstrated that FK506 is a potent regulator of the induction and persistence of the asthmatic inflammatory response.

FK506 inhibits T cell activation by targeting NFAT activation *via* calcium signaling and is widely used as an immunosuppressive agent (31). In this study, we showed that FK506 targets T cell migration into the lungs. T_H_2 cells are essential for the persistence of airway inflammation induced by administration with cysteine protease allergen, and IL-13 is a crucial cytokine in controlling the recruitment of T1/ST2^+^ and GATA3^+^ T_H_2 cells to the inflammatory site. The induction of T_H_2 cells is critical for the persistence and exacerbation of airway inflammation (11, 12). Thus, FK506 is a potent inhibitor of the IL-13-dependent T_H_2 cell recruitment induced by the nasal administration of a protease allergen.

On the other hand, FK506 was also effective in inhibiting eosinophilia during the induction phase, which is mainly controlled by ILC2 cells. Recently, Kandikattu K et al. reported that FK506 can reduce eosinophil infiltration by downregulation of calcineurin activity in the lung tissue from Aspergillus fumigatus challenged mice (23). However, eosinophil was not a direct target of FK506 in their model. Our data indicated that ILC2s were a target of FK506 in the papain induced asthmatic responses. It has been reported that FK506-sensitive calcium signaling is dispensable for IL-33-dependent ILC2 activation (32). Indeed, our data indicated that IL-33-induced airway inflammation was FK506-resistant (**Figure 6**). Previous evidence indicated that other calcium-dependent signaling molecules, lipid mediators, and NMU can also control ILC2 cell activation (24, 32). Our data suggest that the LTB_4_ signal could be a possible target of FK506 in the ILC2 activation process. Locksley et al. reported that cyclosporine A, another calcineurin inhibitor, reduced IL-13 production in cultured ILC2 cells stimulated with LTB_4_ (24). Therefore, FK506-sensitive calcineurin-mediated ILC2 activation is critical for protease-mediated airway inflammation.

Eosinophils are the primary effector cells involved in asthmatic airway inflammation (10, 33). Several previous reports have indicated that blocking GM-CSF signaling promotes the inhibition of type 2 inflammatory responses (34–38). Here, we found that FK506 suppressed GM-CSF production in ILC2 cells. Thus, the inhibition of ILC2-derived GM-CSF may be a possible mechanism of the FK506-mediated suppression in the protease-mediated airway inflammation during the induction phase.

In conclusion, we demonstrated that T_H_2 and ILC2 cells could be therapeutic targets for FK506 in type 2 airway inflammation. FK506 inhibits the differentiation of T_H_2 cells, which causes chronic inflammation by attenuating ILC2 function. Since ILC2 and T_H_2 cells are involved in allergic airway inflammation, such as asthma, our results strongly support the potential clinical value of FK506 for these type 2 inflammatory diseases. The inhibitory mechanism of FK506 in a cysteine protease allergen-induced allergic mouse model sheds new light on future therapeutic strategies for asthma.

## Methods

### Mice

C57BL/6Jjcl mice were purchased from CLEA (Meguro, Tokyo, Japan). Six- to ten-week-old female mice were used in the experiments. *Cd3e^-/-^* and *Il13^tomato^* mice were kindly provided by Dr. Bernard Malissen (Aix Marseille Université, Marseille, France) and Dr. Andrew NJ McKenzie (MRC Laboratory of Molecular Biology, Cambridge, UK) (39, 40).

All transgenic mice were obtained from a C57BL/6 background. All mice were maintained under specific pathogen-free conditions, and animal care was performed according to the guidelines of the RIKEN Yokohama Institute.

### Cysteine protease allergen-induced and IL-33-induced allergic mouse model

Mice were intranasally administered with papain (50 µg/head/day; Sigma-Aldrich, St. Louis, MO, USA) or IL-33 (1 µg/head/day; Biolegend, San Diego, CA, USA) for three days. The lungs and bronchoalveolar lavage (BAL)s of the treated mice were harvested on days 1 and 7. The harvested lung tissues were roughly chopped with a Gentle MACS Dissociator (Miltenyi Biotec) and digested with 5 mL of HBSS (Thermo Fisher, Waltham, MA, USA) containing DNase I (75 µg/mL) and collagenase D (400 U/mL) for 30 min at 37°C. The lung homogenate was ground with a Gentle MACS Dissociator, passed through a 100-µm cell strainer (BD Biosciences, Franklin Lakes, NJ, USA), and fractionated with 30% Percoll (GE Healthcare, Uppsala, Sweden). The cell pellet was then treated with an RBC lysis buffer (Biolegend, San Diego, CA, USA) and suspended in MojoSort buffer (Biolegend). After the combining of BAL and lung isolated cells, eosinophils were analyzed using anti-Siglec-F, anti-Gr-1, and anti-CD11c antibodies after eliminating alveolar macrophages with anti-F4/80 antibodies (**Figure 1**).

For chemical treatment, the mice were intraperitoneally treated with FK506 (0.2 mg/head/day; Cayman Chemical, Ann Arbor, MI, USA) or Dexamethasone (0.1 mg/head/day; Sigma-Aldrich, St. Louis, MO, USA) for six days. These mice were then administered with papain intranasally in the last three days.

### Histology

The lungs were fixed with paraformaldehyde (4%) and frozen in OCT compound (Sakura Finetek, Tokyo, Japan). Sections (5 μm) were stained with an HE or PAS Staining Kit (Muto Pure Chemicals, Tokyo, Japan), or anti-CD4 antibody (clone: RM4-5; 1:50, Biolegend, 100506). Images were acquired using a BZ-X700 microscope (Keyence). Two regions of interest (ROIs) were set on each section, and the numbers of acidic mucus-positive goblet cells and infiltrating lymphocytes were counted.

### Flow cytometry

Cell staining was performed using antibodies against B220 (clone: RA3-6B2; used at 1:500, Biolegend, 103227), CD3e (clone:145-2C11; 1:500, Biolegend, 100304), CD4 (clone: GK1.5; 1:500, Biolegend, 100423), CD5 (clone:53-7.3; 1:200, Biolegend, 100604), CD8a (clone:53-6.7; 1:500, Biolegend, 100704), CD11b (clone: M1/70; 1:200, Biolegend, 101204), CD11c (clone: N418; 1:200, Biolegend, 117304), CD45.2 (clone:104; 1:500, eBioscience, 11-0454-85), CD49b (clone: DX5; 1:200, Biolegend, 108904), F4/80 (clone: BM8; 1:200, Biolegend, 123106), FceR1 (clone: Mar1; 1:200, Biolegend, 134318), Gr-1 (clone: RB6-8C5; 1:500, BD Biosciences, 553124), NK1.1 (clone: PK136; 1:300, Biolegend, 108704), Siglec-F (clone: E50-2440; 1:400, BD Biosciences, 552126), Thy1.2 (clone:30-H12; 1:500, BD Biosciences, 105324), Podoplanin (clone:8.1.1; 1:100, Biolegend, 127410), and Ter119 (1:200, eBioscience, 13-5921-82). Flow cytometric analysis and cell sorting were performed using FACSCalibur and FACSAria III systems (BD Biosciences), and data were analyzed using FlowJo software (BD Biosciences). Doublet cells were excluded by FL-2A/FL-2H plots, then FSC/SSC plots were used to narrow down eosinophil or T cell populations followed by further gating with Siglec-F/Gr-1 or Siglec-F/CD11c (for eosinophils), or CD4/ST-2 (for T_H_2 cells) plots (**Supplementary Figure 3**).

### RNA-seq analysis

Total RNA was isolated from freshly sorted lung epithelial cells, basophils, and ILC2 using the TRIzol reagent. The 3’ mRNA-seq Library Prep Kit (Lexogen, Vienna, Austria) was used for constructing sequencing libraries. RNA libraries were prepared for sequencing using the standard Lexogen 3’ QuantSeq protocols. After sequencing using the HiSeq 1500 platform (Illumina, San Diego, CA, USA), sequenced reads were trimmed for adaptor sequences, masked for low-complexity or low-quality sequences, and mapped to the whole mouse genome (mm10) using STAR 2.7.0 c (41).

### Cytokine analysis in ILC2 cells

Lineage-positive lung CD45^+^ cells were eliminated using lineage markers (CD3e, CD4, CD5, CD8a, CD11c, CD19, F4/80, Ly-6G, and NK1.1), and Thy1.2^+^ cells were isolated as ILC2 cells. To assess the levels oILC2-derived cytokines in papain-treated mice, isolated ILC2 cells were cultured in RPMI medium containing 10 ng/mL of recombinant murine IL-7 (rIL-7) (Peprotech, Cranbury, NJ, USA) for 12 h, and cytokine and chemokine levels in the supernatant were measured using a MAGPIX Multiplexing System (Luminex, Austin, TX, USA) and a MLLIPLEX Mouse High Sensitivity T Cell Panel (Merck, Darmstadt, Germany).

To investigate the role of calcium signaling in ILC2 cells, isolated ILC2 cells were stimulated with 10 ng/mL of recombinant murine IL-33 (Biolegend), 30 ng/mL of PMA (Sigma-Aldrich), 500 ng/mL of ionomycin (Sigma-Aldrich), or 10 nM of LTB_4_, LTC_4_, LTD_4_, or LTE_4_ (Cayman Chemical) in the presence of 10 ng/mL of rIL-7 (Peprotech) for 40 h. Stimulation was performed with or without FK506 (1000 nM). Cytokine levels were determined as previously described (25).

### Statistical analyses and reproducibility

Statistical comparisons between groups were performed using Prism software version 8.0.2 (Graph Pad Software, San Diego, CA, USA). Data are presented as the mean ± SEM. Statistical analyses were performed using the Mann-Whitney U test (*p<0.05, **p<0.01).

## Data availability statement

The datasets presented in this study can be found in online repositories. The names of the repository/repositories and accession number(s) can be found in the article/**Supplementary Material**.

## Ethics statement

RIKEN Yokohama Institutional Animal Care Committee reviewed and approved the animal study.

## Author contributions

MK designed and conceptualized the research; CT, NS, YS, and KM performed the mouse experiments; CT and SK performed the histological analysis; CT, NS, and KM performed RNAseq analysis; KM, HM, YM, and MK prepared the manuscript. All authors contributed to the article and approved the submitted version.

## Funding

This work was supported by a collaborative grant from Maruho Co., Ltd. The funder was not involved in the study design, collection, analysis, interpretation of data, the writing of this article or the decision to submit it for publication.

## Acknowledgments

We thank Dr. Mallissen for providing *Cd3e^-/-^* mice and Dr. McKenzie for providing *Il13^tomato^* mice.

## Conflict of interest

HM and YM are employees of Maruho Co. Ltd.

The remaining authors declare that the research was conducted in the absence of any commercial or financial relationships that could be construed as a potential conflict of interest.

## Publisher’s note

All claims expressed in this article are solely those of the authors and do not necessarily represent those of their affiliated organizations, or those of the publisher, the editors and the reviewers. Any product that may be evaluated in this article, or claim that may be made by its manufacturer, is not guaranteed or endorsed by the publisher.
