# Supplementary figures and images for: Role of FK506-sensitive signals in asthmatic lung inflammation
Source: Front Immunol. 2022 Nov 9;13:1014462. doi: 10.3389/fimmu.2022.1014462 (PMC9683035; doi:10.3389/fimmu.2022.1014462)

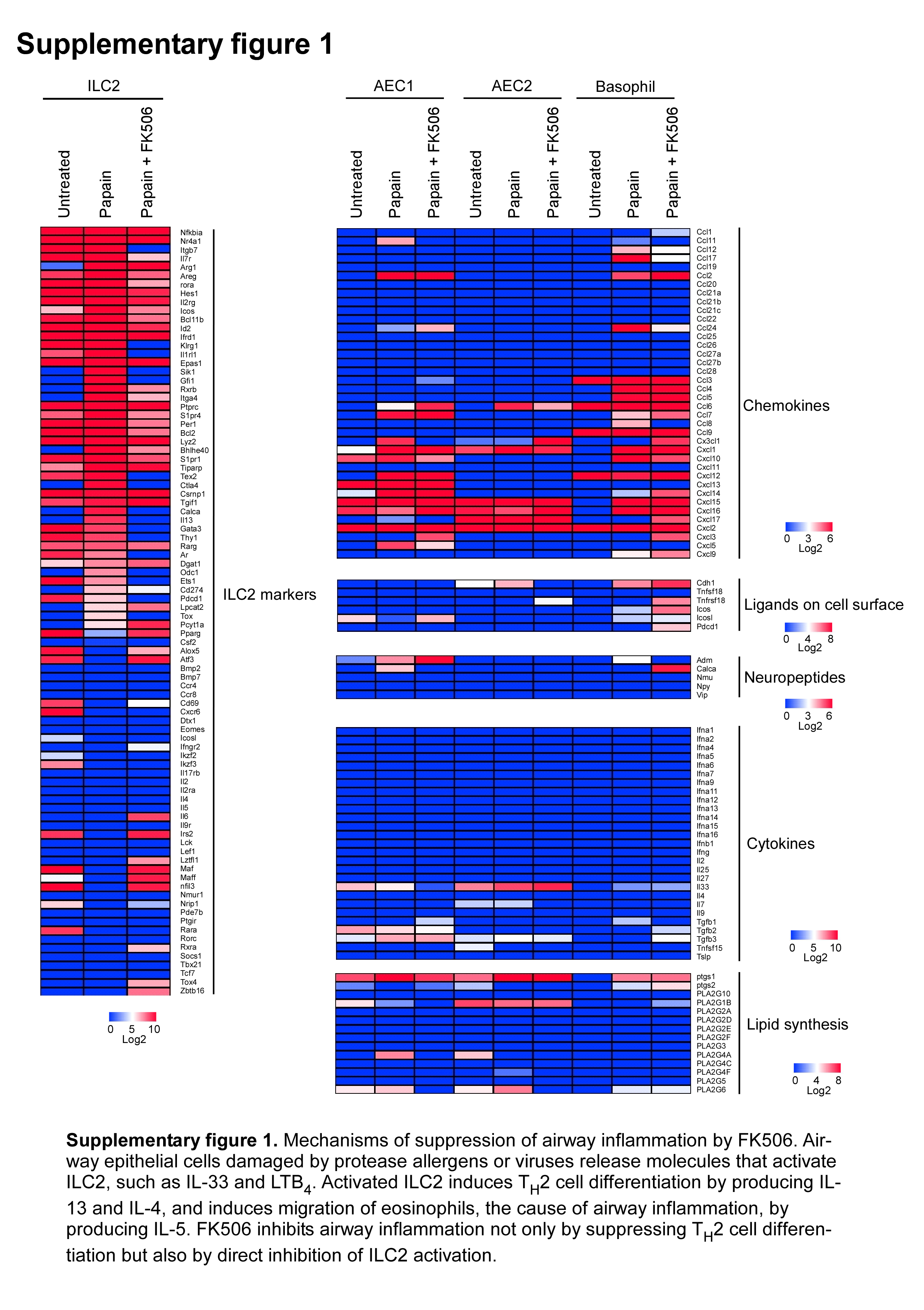

Supplement: Supplementary file 1 [file Image_1.tif]

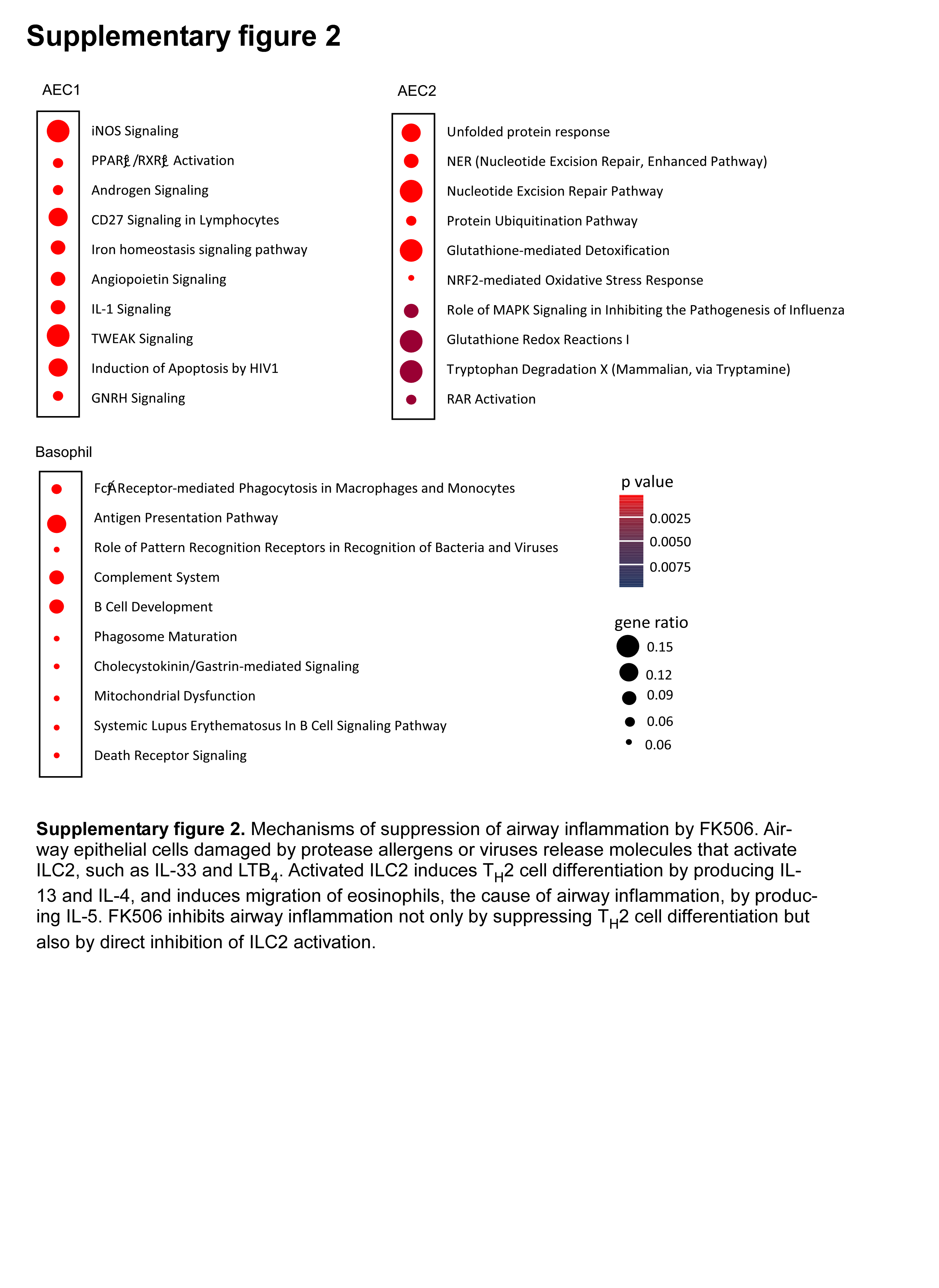

Supplement: Supplementary file 2 [file Image_2.tif]

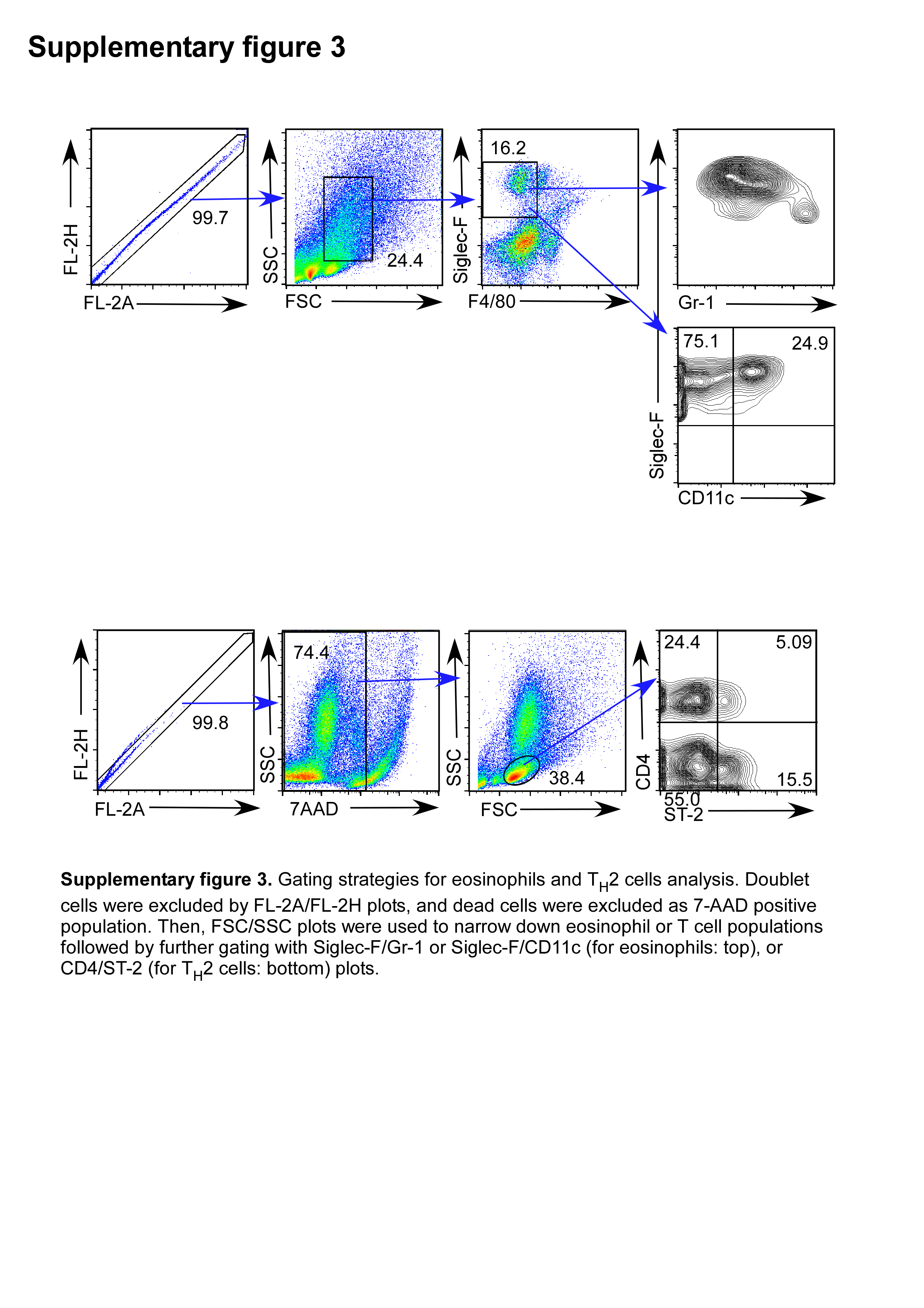

Supplement: Supplementary file 3 [file Image_3.tif]
